# Animated Videos as a Tool for Teaching Uncommon ENT Diagnoses

**DOI:** 10.7759/cureus.63283

**Published:** 2024-06-27

**Authors:** Aadil Rahman, Douglas Pereira Nanu, Jyoti M Sharma, Ryan Nagy, Michele M Carr

**Affiliations:** 1 Pediatric Emergency Medicine, Jacobs School of Medicine and Biomedical Sciences, University at Buffalo, Buffalo, USA; 2 Otolaryngology, Washington State University, Spokane, USA; 3 Otolaryngology, Jacobs School of Medicine and Biomedical Sciences, University at Buffalo, Buffalo, USA; 4 Emergency Medicine, Memorial Health Systems, Marietta, USA

**Keywords:** resident teaching, medical education, emergency department, emergency medicine, nasal septal hematoma

## Abstract

Objective: The purpose of this study was to evaluate the knowledge of healthcare professionals and learners regarding the diagnosis and management of nasal septal hematomas (NSH). The secondary objective was to evaluate the effectiveness of a short-form animated video as an educational tool.

Methods: A cross-sectional survey study of healthcare professionals and medical students in the United States was undertaken from October 2022 to June 2023. A pre-test survey was distributed to assess participants’ baseline knowledge of NSH management. An educational video on nasal septal hematoma management was presented, followed by a post-test survey to measure the effectiveness of the video.

Results: A total of 142 participant results were collected, 62 (43.7%) of which were attending physicians. There was a significant improvement in knowledge scores across the sample, with a median pre-test score of 83.0% (interquartile range (IQR) 33) and a median post-test score of 100.0% (IQR 17, p<0.001). Additionally, on a visual analog scale (VAS), comfort levels in managing NSH improved from 3.20 to 4.82 (p<0.001) for the entire sample.

Conclusion: NSH is a rare yet potentially devastating otolaryngologic emergency that requires prompt diagnosis and management. A short-form animated video can be an effective tool for educating emergency professionals on diagnosing and managing NSH.

## Introduction

Nasal injuries are common during facial trauma, especially in pediatric and adolescent patients. Septal injury is more common in younger patients due to softer septal cartilage, making it more prone to buckling [1,2]. Significant injury to the nasal septum can result in a nasal septal hematoma (NSH), which carries significant morbidity if missed, misdiagnosed, or mismanaged. Although rare, NSH has been reported to occur in 0.8%-1.6% of patients with nasal injuries who seek care from an otolaryngologist. However, this incidence is an estimate, and the exact number remains unknown [3].

Nasal septal hematoma most commonly occurs secondary to traumatic injury, including sports injuries, vehicular accidents, falls, physical assault, occupational injuries, or any physical disturbance to the nose [3]. The septal cartilage receives nutrients through diffusion from the overlying perichondrium, supplied by Kiesselbach’s plexus. Traumatic injuries can disrupt and tear the submucosal vasculature, resulting in blood collection between the cartilage and mucoperichondrium. This hematoma formation creates a barrier and impairs the diffusion of nutrients from the mucoperichondrium to the inherently avascular cartilage, which can lead to necrosis of the cartilage. If the septum is fractured, blood can cross the septal plane, resulting in a bilateral septal hematoma. Disruption of the septal vasculature bilaterally can further hasten septal necrosis. If untreated, this may eventually result in a nasal septal abscess, septal perforation, or saddle-nose deformity [4].

A prompt, accurate diagnosis and management of NSH is required to prevent significant morbidity and disfigurement. Achieving this objective, however, remains challenging. Ali et al. demonstrated the frequency with which the diagnosis of NSH is missed. In this single-center electronic health record retrospective chart review of 2762 patients diagnosed with nasal trauma, 13 (0.4%) were also diagnosed with NSH. Eleven out of the 13 patients required at least two visits to healthcare providers before the diagnosis was appropriately made. On only two occasions was the diagnosis correctly made on the initial evaluation. The diagnosis was missed across the healthcare spectrum, including in the emergency department (ED), primary care clinics, urgent care clinics, and otolaryngology clinics. In total, seven (54%) out of the 13 patients developed long-term complications [5].

Due to the relative rarity of acute NSH, healthcare providers may be uncertain about the diagnosis or management of the condition. To educate medical providers on this topic, we developed an educational video that comprehensively covers the proper diagnosis and management of NSH. Our primary goal is to reduce the occurrence of missed diagnoses and ultimately prevent permanent morbidity resulting from such oversights. Therefore, the purpose of this study was to evaluate the knowledge of a broad spectrum of medical providers regarding the management of NSH, an uncommon ENT diagnosis, and subsequently enhance their knowledge through the animated educational video. Additionally, we envision the potential of these institution-made educational videos in the future to serve as valuable resources for training healthcare providers in managing other uncommon medical problems that require immediate attention.

## Materials and methods

Selection and survey criteria

Anonymous survey data samples were collected via Google Forms from October 2022 until June 2023. A convenience sample of Emergency Medicine attendings, fellows, residents, advanced practice providers (physician assistants and nurse practitioners), and medical students (MS) was recruited from across the country via local recruitment, email, and listservs. The level and type of medical training of the participants were recorded. The participants were asked to complete an eight-question pre-test, then watch a five-minute YouTube video created by the research team, and immediately conclude with a seven-question post-test. They also included a seven-point Likert scale assessing their comfort managing NSH. Higher scores indicated higher levels of comfort. Only the pre-test contained a question asking if the respondent had managed an NSH before. The test questions were formulated based on several essential factors crucial for diagnosing NSH. Participants were queried about their past experiences in diagnosing and treating NSH, as well as their confidence level in managing such cases. Questions included the context in which septal hematomas can occur, the potential complications associated with them, and the cardinal signs indicative of nasal septal hematomas. Finally, participants were asked about the appropriate management of nasal septal hematomas, discharge instructions to provide, and the course of action to be taken in the event of bilateral NSH. Questions were validated for accuracy by the Otolaryngology department at the University at Buffalo in Buffalo, NY. The YouTube video was a low-cost animation made without a media expert's contribution. The YouTube video is available in the following link: https://www.youtube.com/watch?v=ZW8DxrgNNno. Pre- and post-test questionnaire can be found via the following link: https://forms.gle/pzS1tgkxU8eXdGGm9. 

Data analysis

For the purpose of comparison, the investigated subjects were grouped based on their level of medical training: attendings, trainees, advanced practice providers (APPs), medical students, and others. Additionally, the investigated subjects were further divided into subgroups to indicate their area of expertise and specialization.

The analysis of categorical demographic data as well as continuous measures (means, medians, standard deviations, and interquartile ranges) was performed using IBM SPSS Statistics, Version 29 for MacOS (IBM Corp, Armonk, NY, USA). Comparison groups were assessed for normal distribution using the Kolmogorov-Smirnov and Shapiro-Wilk tests. If the P value was <0.05, the data was considered not normally distributed. In cases where the data did not follow a normal distribution, the Wilcoxon signed rank test was employed, and results were reported using median and interquartile range (IQR) values. With the Wilcoxon signed rank test if the medians are equal but the distributions of the two groups differ in other ways (such as variance or shape), the test may still indicate a significant result. For example, if one group has a more concentrated distribution around the median while the other group has a more spread-out distribution, leading to differences in the ranks of the paired observations. For groups with a normal distribution, paired t-tests were conducted, and results were presented using mean and standard deviation values.

The null hypothesis in this study posited that no difference existed between pre-test and post-test scores after viewing the YouTube video on how to properly manage NSH. An additional objective was to ascertain whether there was an increase in the comfort level of survey respondents in managing NSH before and after watching the YouTube video. For all reported P values, statistical significance was set at a P value of ≤0.05.

## Results

The survey was anonymized and carried out through Google Forms. The educational video was hosted on YouTube and a link was embedded into the survey. In total, 142 participants completed the study. Sixty-two (43.7%) were attendings, 38 (26.8%) were trainees, who were either fellows or residents, 5 (3.5%) were APPs, and 31 (21.8%) were medical students. Six (4.2%) were categorized as Other, meaning they did not fall into any of the categories previously mentioned. Thirty-four (23.9%) had diagnosed NSH in the past, 26 (76.5%) of whom were attendings (Table 1).

**Table 1:** Participant Demographics NSH=Nasal septal hematoma.

Training Level	Number	(%)	Prior NSH Diagnosis
Attendings	62	43.7	26
Pediatrics	1	0.7	0
Pediatric Emergency Medicine	53	37.3	23
Emergency Medicine	8	5.6	3
Trainees	38	26.8	3
Pediatric Fellows	7	4.9	0
Emergency Medicine Fellows	8	5.6	1
Pediatric Residents	5	3.5	0
Emergency Medicine Residents	17	12.0	2
Family Medicine Resident	1	0.7	0
Advanced Practice Providers	5	3.5	2
Physician Assistants	4	2.8	1
Nurse Practitioner	1	0.7	1
Medical Students	31	21.8	0
Other	6	4.2	2
Total	142	100.0	34

There was a significant improvement in knowledge scores after the educational video across the sample, with a median pre-test score of 83.0% (IQR 33) and a median post-test score of 100.0% (IQR 17, p<0.001). Pre-test and post-test scores for attending, trainee, and medical student subgroups were 100% (IQR 17) and 100% (IQR 17, p=0.045), 83% (IQR 16) and 100% (IQR 17, p<0.001), and 67% (IQR 33) and 83% (IQR 33, p<0.001), respectively. Within the trainee subgroup, only Emergency Medicine Residents showed statistically significant improvement with pre-test scores of 67% (IQR 16) and post-test scores of 100% (IQR 17, p<0.001) (Table 2).

**Table 2:** Test Score Analysis Before and After Educational Intervention *P values in bold indicate statistical significance. †Central tendency reported as mean (SD) due to normal distribution. Test statistic value dependent on whether central tendency was reported as mean with SD (t-value) or if it was reported as median with IQR (z-statistic).

Training Level (n)	Pre-Test Median % (IQR)	Post-Test Median % (IQR)	Test Statistic	p value*
Attendings (62)	100.0 (17)	100.0 (17)	2.005	0.045
Pediatrician (1)	-	-	-	-
Pediatric Emergency Medicine (53)	83.0 (17)	83.0 (17)	1.857	0.07
Emergency Medicine (8)	100.0 (17)	100.0 (12.8)	1.00	1.00
Trainees (38)	83.0 (16)	100.0 (17)	4.417	<0.001
Pediatric Fellows (7)	83.0 (17)	83.0 (17)	1.633	0.25
Emergency Medicine Fellows (8) †	91.5 (9.1)	89.5 (12.4)	0.403	0.70
Pediatric Residents (5) †	53.2 (18.2)	83.4 (23.5)	-2.463	0.07
Emergency Medicine Residents (17)	67.0 (16.0)	100.0 (17.0)	2.996	<0.001
Family Medicine Resident (1)	-	-	-	-
Advanced Practice Providers (5)	83.0 (42)	100.0 (0)	-1.645	0.10
Physician Assistants (4) †	79.9 (31.7)	100.0 (0)	-1.325	0.28
Nurse Practitioner (1)	-	-	-	-
Medical Students (31)	67.0 (33)	83.0 (33)	3.930	<0.001
Other (6)	75.0 (37.3)	100.0 (21)	-1.941	0.11
Total (142)	83.0 (33)	100.0 (17)	6.162	<0.001

The comfort in managing NSH improved from 3.20 to 4.82 for the entire sample (p<0.001). All subgroups showed a statistically significant improvement in comfort in managing NSH except for Other (Table 3).

**Table 3:** Comfort Level Analysis Before and After Educational Intervention *P values in bold indicate statistical significance.

Training Level (n)	Pre-Test Comfort Level Mean (SD)	Post-Test Comfort Level Mean (SD)	t-value	p value*
Attendings (62)	4.37 (1.54)	5.37 (1.07)	5.940	<0.001
Trainees (38)	2.76 (1.50)	4.79 (1.32)	6.270	<0.001
Advanced Practice Providers (5)	2.20 (0.84)	4.60 (1.14)	2.134	0.04
Medical Students (31)	1.61 (1.02)	3.9 (1.54)	9.760	<0.001
Other (6)	2.83 (2.56)	4.17 (2.14)	1.476	0.11
Total (142)	3.20 (1.82)	4.82 (1.41)	4.620	<0.001

## Discussion

According to the Centers for Disease Control and Prevention (CDC), EDs in the United States care for approximately 38 million injury-related concerns annually. Data specifically regarding nasal or facial injuries are unlisted; however, injuries to the head account for 5.7 million visits, which makes up approximately 15% of all injury-related concerns [6]. While many nasal injuries may not raise immediate concern, NSH stands as an exception, highlighting the significance of accurate identification and management [7]. Previous research has shown that urgent otolaryngology consultation is available at numerous EDs across the country; however, this is impractical for certain EDs due to myriad reasons, including financial limitations [8]. As emergency providers are typically the point of first contact for these injuries, it is crucial for them to be well-versed in managing them, including nasal or facial trauma.

In the realm of medical education, the efficacy of visual learning materials and digital media has been substantiated, proving instrumental in the education of healthcare practitioners [9,10]. Video learning has only gained traction in recent years, and a plethora of video-based teaching modalities are now accessible. While long-form videos have traditionally been used for comprehensive chapter reviews and board examination preparations, the ubiquity of Internet access and the surge in social media platforms have engendered novel avenues such as YouTube, Twitter, Instagram, and TikTok. These platforms can be potent mediums for dispensing concise yet impactful educational content. "Just-in-time" videos, a novel concept in medical education, have recently emerged, demonstrating success in training providers for precise skills or patient management techniques immediately prior to their real-time application. This innovative approach has demonstrated its efficacy in improving the acquisition and application of clinical proficiencies [11]. These short-form video methodologies enable learners to autonomously revisit topics, accommodating their schedules and learning pace, thereby fostering repetitive learning. Most importantly, these resources can play a pivotal role in addressing infrequent yet high-stakes scenarios such as NSH, which necessitate urgent and accurate intervention to ensure optimal patient outcomes.

Limitations

Limitations inherent to our study include a relatively small sample size, particularly notable in the case of APPs, family medicine residents, and pediatricians. For each of these cohorts, statistical power fell below the established threshold of 0.800. Therefore, in these cohorts, the available sample sizes did not suffice to unequivocally discount the possibility that observed discrepancies are a result of chance-induced sampling fluctuations. This renders us unable to dismiss the hypothesis that the population mean of pre-test samples equals or exceeds that of post-test samples within these specific cohorts. Therefore, no definite conclusions can be made within those cohorts.

The inherent susceptibility of convenience samples to selection and sampling biases further compounds the limitations of our investigation. Another notable constraint of our study lies in its inability to address the matter of long-term knowledge retention. To circumvent this limitation, a potential avenue for subsequent research involves conducting a secondary study that evaluates knowledge retention by means of a post-test administered after a specified time delay.

## Conclusions

Improvements in knowledge and comfort in managing an NSH were demonstrated in most analyzed groups. Our data demonstrates that a short-form questionnaire and educational video can be an effective teaching tool for healthcare providers across all disciplines. The landscape of medical education has undergone a revolution over the past two decades and will continue to transform in the years to come. Embracing this evolving landscape will be pivotal in shaping the education of future learners and medical professionals for generations to come.
